# TLR7 Mediates HIV‐1 Tat‐Induced Cellular Senescence in Human Astrocytes

**DOI:** 10.1111/acel.70086

**Published:** 2025-04-30

**Authors:** Neda Rezagholizadeh, Gaurav Datta, Wendie A. Hasler, Erica C. Nguon, Elise V. Smokey, Xuesong Chen

**Affiliations:** ^1^ Department of Biomedical Sciences University of North Dakota School of Medicine and Health Sciences Grand Forks North Dakota USA

**Keywords:** astrocyte, endolysosomes, HIV‐1 tat, senescence, TLR7

## Abstract

Cellular senescence contributes to accelerated aging, neuroinflammation, and the development of HIV‐associated neurocognitive disorders (HAND) in the era of combined antiretroviral therapy (cART). One HIV viral factor that could lead to cellular senescence is the persistence of HIV‐1 Tat in the brain. As a secreted viral protein, Tat is known to enter endolysosomes of cells through receptor‐mediated endocytosis, and we have shown that Tat induces endolysosome damage and dysfunction. Significantly, endolysosome dysfunction has been strongly linked to cellular senescence. However, it is not known whether endolysosome dysfunction represents a driver or consequence of cellular senescence. Because Tat‐induced endolysosome damage represents an early step in exogenous Tat‐induced cellular senescence, we tested the hypothesis that Tat induces cellular senescence via an endolysosome‐dependent mechanism in human astrocytes. We demonstrated that Tat interacts with an endolysosome‐resident Toll‐like receptor 7 (TLR7) via its arginine‐rich basic domain, and such an interaction results in endolysosome damage and the development of a senescence‐like phenotype including cell cycle arrest, enhanced SA‐β‐gal activity, and increased release of senescence‐associated secretory phenotype (SASP) factors (IL‐6, IL‐8, and CCL2). Thus, our finding provided mechanistic insights whereby Tat induces endolysosome damage and cellular senescence in human astrocytes. We provide compelling evidence that endolysosome damage drives the development of cellular senescence. Our findings also highlight the novel role of TLR7 in the development of cellular senescence and suggest that TLR7 represents a novel therapeutic target against senescence and the development of HAND.

## Graphical abstract

Once internalized into endolysosomes, HIV‐1 Tat interacts and activates TLR7, causing endolysosome damage. Such interaction between HIV‐1 Tat and TLR7 also leads to a senescence‐like phenotype in astrocytes, including cell cycle arrest, elevated levels of p21 and p16 proteins, increased SA‐β‐gal activity, and enhanced secretion of SASP factors.

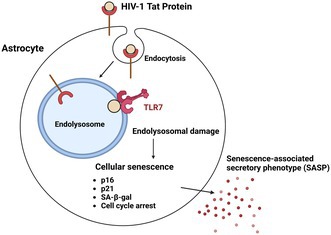

## 1 Introduction

Combined antiretroviral therapy (cART) has dramatically increased the lifespan of people with HIV‐1 (PWH) (May et al. 2014). Currently, more than half of the PWHs are aged 50 and older in the US (High et al. 2012), and this number will increase to more than three quarters by 2030 (Smit et al. 2015). Advanced age increases the risk for neurocognitive impairment (Alford and Vera 2018; Bhaskaran et al. 2008), and 50% of PWH develop HIV‐associated neurocognitive disorders (HAND) even with cART (Wang et al. 2020). Although the underlying mechanisms remain unclear, chronic neuroinflammation (McArthur and Johnson 2020), accelerated aging (Breen et al. 2022), and neurodegeneration (Ellis et al. 2007) play a critical role in the pathogenesis of HAND. Emerging evidence indicates that the HIV‐1 brain exhibits molecular signatures of brain aging (Cole et al. 2017) and cellular senescence (Hove‐Skovsgaard et al. 2020; Hsiao et al. 2021; Montano et al. 2022). Besides the degenerating nature of cellular senescence, senescent cells secrete pro‐inflammatory and pro‐oxidative factors called senescence‐associated secretory phenotype (SASP) that could elicit deleterious paracrine‐like effects on neighboring cells, contributing to brain aging, neuroinflammation, and neurodegeneration (Holloway et al. 2023; Meldolesi 2023).

The development of HAND in the ART era has been attributed, at least in part, to the persistence of HIV‐1 Tat, an essential factor for viral transcription (Kameoka et al. 2002) that is actively secreted from HIV‐1 infected cells (Agostini et al. 2017; Chang et al. 1997; Ensoli et al. 1990; Rayne et al. 2010). Significantly, current anti‐HIV strategies do not block the secretion of Tat (Mediouni et al. 2012), and brain levels of Tat remain elevated even when HIV‐1 levels are below detectable levels (Henderson et al. 2019; Johnson et al. 2013). In the brain, Tat could exert a direct neurotoxic effect (Bertrand et al. 2014; Fitting et al. 2013; Hargus and Thayer 2013; Nath and Steiner 2014) or induce neuroinflammation by interacting with astrocytes (Blanco et al. 2008; Conant et al. 1998; El‐Hage et al. 2005; Kutsch et al. 2000; Nath et al. 1999; Priyanka et al. 2020; Tewari et al. 2015; Williams et al. 2009), macrophage/microglia (Eugenin et al. 2005; Periyasamy et al. 2019; Thangaraj et al. 2018), and other CNS cells (Ajasin and Eugenin 2020; Marino et al. 2020). Furthermore, Tat has been shown to induce cellular senescence in microglia (Thangaraj et al. 2021), mesenchymal stem cells (Beaupere et al. 2015), astrocytes (Pillai et al. 2023), and endothelial cells (Hijmans et al. 2018; Zhan et al. 2016). Such Tat‐induced cellular senescence in CNS cells could contribute to the development of accelerated aging, neuroinflammation, and neurodegeneration in HAND (Cole et al. 2017; Dickens et al. 2017; Mackiewicz et al. 2019; Zhao et al. 2020, 2022). However, it is not clear how Tat induces cellular senescence.

As secreted viral proteins present in the brain, Tat enters endolysosomes of CNS cells via receptor‐mediated endocytosis (Debaisieux et al. 2012; Frankel and Pabo 1988; Gaskill et al. 2017; Liu et al. 2000; Mann and Frankel 1991; Tyagi et al. 2001), and we have shown that Tat induces endolysosome damage and dysfunction in neurons and astrocytes (Chen et al. 2013; Hui et al. 2012; Khan et al. 2022). Endolysosomes are critical for the degradation of macromolecules or damaged organelles delivered to lysosomes via endocytosis or autophagy and critical for metabolism and cellular homeostasis (Ballabio and Bonifacino 2020). As such, endolysosome dysfunction leads to abnormal accumulation of undegraded materials and enlargement of endolysosomes (Datta et al. 2021; Khan et al. 2022) and augmented release of their luminal contents via exocytosis (Datta et al. 2019; Kim et al. 2021) that contribute to inflammation (Bordon 2011; Qian et al. 2017; Rawnsley and Diwan 2020; Toyama‐Sorimachi and Kobayashi 2021; Yambire et al. 2019). Furthermore, a key feature of cellular senescence is endolysosome dysfunction (Curnock et al. 2023; Gorgoulis et al. 2019; Tan and Finkel 2023), which includes endolysosome enlargement, endolysosome de‐acidification, endolysosome membrane leakage, and increased endolysosome content, with senescence‐associated β‐galactosidase (SA‐β‐gal) being the most widely employed marker of the senescent state. Because Tat‐induced endolysosome damage represents an early step in Tat‐induced cellular senescence, the present studies are aimed at elucidating the extent to which Tat induces inflammatory response and cellular senescence in human astrocytes via an endolysosome‐dependent mechanism.

## 2 Results

### 2.1 The Arginine‐Rich Basic Domain Is Critical for Tat‐Induced Inflammatory Responses and Endolysosome Damage in Human Astrocytes

**FIGURE 1:**
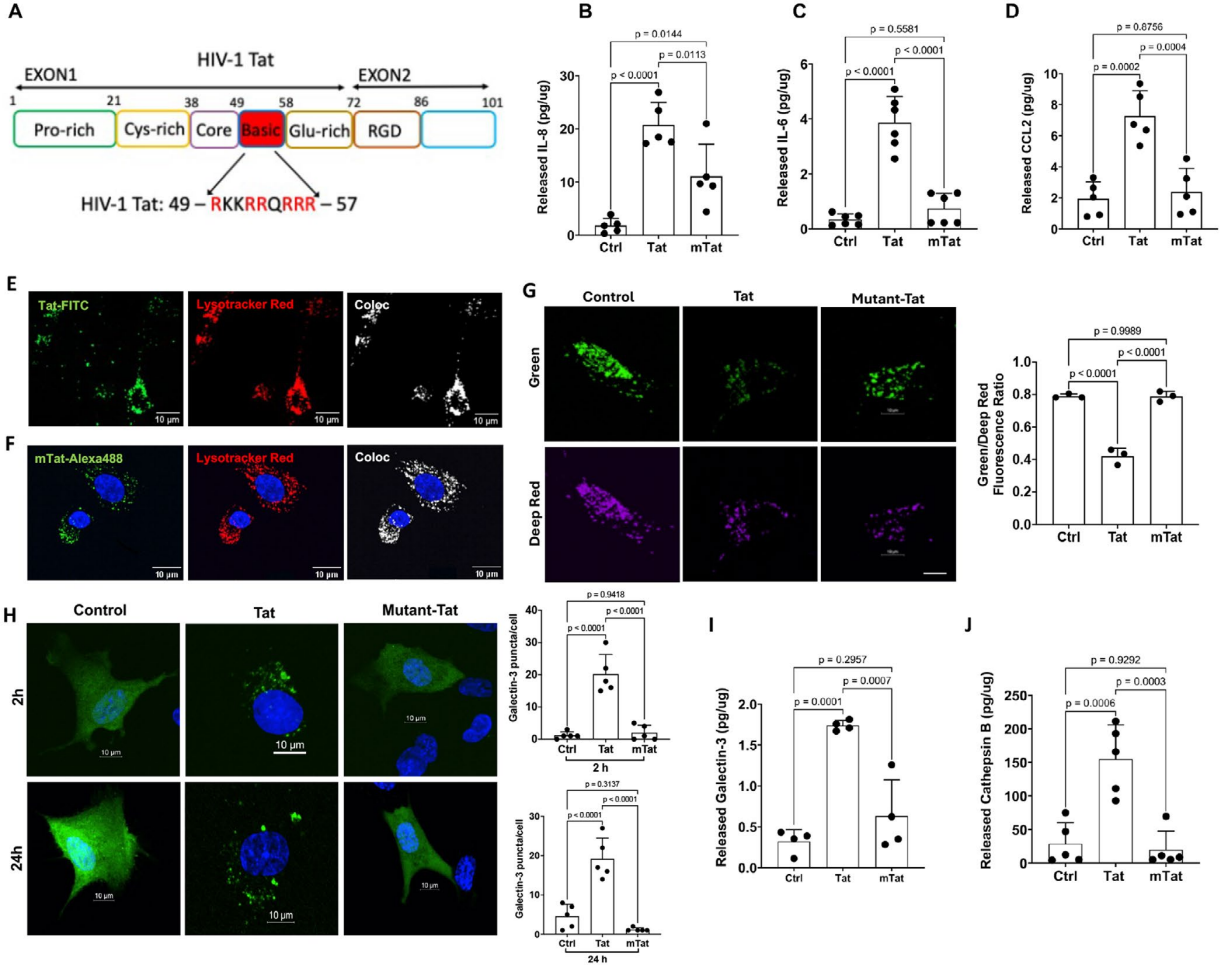
The arginine‐rich domain is critical for Tat‐induced inflammatory response and endolysosome damage in human astrocytes. (A) Schematic of the arginine‐rich basic domain of Tat encompassing amino acids 49–57. (B) Both Tat (100 nM, 48 h) and mutant‐Tat lacking the arginine‐rich domain (100 nM, 48 h) increased levels of IL‐8 in the media of astrocytes; However, Tat‐induced release of IL‐8 is significantly higher that of mutant Tat (*n* = *5*). (C) Tat (100 nM, 48 h), but not mutant‐Tat increased levels of IL‐6 (*n* = *6*). (D) Tat (100 nM, 48 h), but not mutant‐Tat increased levels of CCL2 in the media of astrocytes (*n* = *5*). (E) FITC‐labeled Tat (Tat‐FITC) co‐localized with endolysosomes identified with LysoTracker (red) in human astrocytes. Scale bar = 10 μm. (F) Alexa488 labeled mutant‐Tat (mTat‐Alexa488) co‐localized with endolysosomes identified with LysoTracker (red) in human astrocytes. NucBlue was used for nuclear staining, scale bar = 10 μm. (G) Tat (100 nM for 48 h), but not mutant Tat, induced endolysosome de‐acidification, as indicated by decreased Green/Deep Red fluorescence ratio, scale bar =10 μm (*n* = *3*). (H) In astrocytes expressing EGFP‐tagged Galectin‐3, Tat (100 nM), but not mutant‐Tat (100 nM) significantly increased galectin‐3 puncta formation for 2 h and 24 h treatment (*n* = 5). NucBlue was used for nuclear staining, scale bar =10 μm. (I) Tat (100 nM, 48 h), but not mutant‐Tat, increased levels of galectin 3 in the media of astrocytes (*n* = *4*). (J) Tat (100 nM, 48 h), but not mutant‐Tat, increased levels of cathepsin B in the media of astrocytes (*n* = *5*). Statistics: *N* = Independent replicates. One‐way ANOVA followed by Tukey's post hoc test.

It is known that Tat induces inflammatory responses in various CNS cells including astrocytes (Blanco et al. 2008; Conant et al. 1998; El‐Hage et al. 2005; Kutsch et al. 2000; Nath et al. 1999; Priyanka et al. 2020; Tewari et al. 2015; Williams et al. 2009). Based on our concentration‐ and time‐dependent studies, we demonstrated that Tat (100 nM for 48 h) significantly increased the release of IL‐6, IL‐8, and CCL2 into the media of human astrocytes (Figure 1). At this concentration, Tat failed to increase other inflammatory factors such as IFN‐α, IFN‐β, IFN‐γ, IL‐1α, IL‐18, TNF‐α, IL‐12, and complement C3. At this concentration, Tat did not induce cytotoxic effects as indicated by the LDH release assay (Appendix S1). Although Tat concentrations in brain parenchyma are unknown, nanomolar concentrations of Tat have been detected in CSF of HIV infected individuals on ART drugs (Henderson et al. 2019; Johnson et al. 2013), thus local concentrations of Tat in brain parenchyma could be quite high. Nonetheless, we need a higher concentration of Tat (100 nM) to induce inflammatory responses so that we can confidently assess whether blocking the identified target could attenuate Tat‐induced responses.

The arginine‐rich basic domain of Tat (amino acids 49–57, Figure 1A) has been implicated in several aspects of Tat biology; It is essential for trans‐activating activity and nucleolar localization (Endo et al. 1989), critical for the neurotoxic effect of Tat (Hui et al. 2012; Sabatier et al. 1991; Weeks et al. 1995), and implicated in neuroinflammation (Philippon et al. 1994; Ruiz et al. 2019). Here, we explored the extent to which the mutant‐Tat lacking the arginine‐rich basic domain affects the release of inflammatory factors from astrocytes. We demonstrated that mutant‐Tat (100 nM for 48 h) only significantly increased the release of IL‐8 (Figure 1B), but not IL‐6 (Figure 1C) or CCL2 (Figure 1D). Furthermore, Tat‐induced release of IL‐6, IL‐8, and CCL2 is significantly higher than those induced by mutant‐Tat (Figure 1B–D). At the concentrations used, mutant HIV‐1 Tat did not induce cytotoxic effects in human astrocytes as indicated by the LDH release assay (Appendix S1). Thus, our findings suggest that the arginine‐rich basic domain is critical for Tat‐induced inflammatory responses in human astrocytes.

Increasing evidence indicates that endolysosomes play a critical role in inflammatory responses (Bordon 2011; Qian et al. 2017; Rawnsley and Diwan 2020; Toyama‐Sorimachi and Kobayashi 2021; Yambire et al. 2019). Interestingly, endolysosomes have been involved in the production of cytokines such as IL‐6 (Manderson et al. 2007) and chemokines including IL‐8 (Ohashi et al. 2003) and CCL2 (Wilkinson et al. 2015). Thus, Tat‐induced release of inflammatory factors (IL‐6, IL‐8, CCL2) from human astrocytes could be endolysosome‐dependent. Secreted Tat is known to enter cells through receptor‐mediated endocytosis (Vendeville et al. 2004), with the help of various receptors including HSPGs (Tyagi et al. 2001), CD26 (Gutheil et al. 1994), LRP1 (Liu et al. 2000), CXCR4 (Xiao et al. 2000), and integrin (Barillari et al. 1993). Among these receptors, only HSPGs interact with Tat via the arginine‐rich basic domain (Barillari and Ensoli 2002). Using FITC‐labeled Tat (FITC‐Tat), we demonstrated that Tat enters endolysosomes, as identified with LysoTracker, in human astrocytes (Figure 1E). Mutant‐Tat lacking the arginine‐rich basic domain can also interact with various cell surface receptors including CD26, LRP1, CXCR4, and integrin, all of which could mediate its endocytosis. Using Alexa‐488 labeled mutant‐Tat (mTat‐Alexa488), we investigated the extent to which mutant‐Tat enters endolysosomes of human astrocytes. We observed a significant intracellular presence of mTat‐Alexa488, which co‐localized with endolysosomes identified with LysoTracker (Figure 1F). Thus, the arginine‐rich domain is not essential for the internalization of Tat in human astrocytes.

We have previously demonstrated that Tat enters endolysosomes and induces endolysosome dysfunction in both neurons (Chen et al. 2013; Hui et al. 2012) and astrocytes (Khan et al. 2022). In this study, we investigated the role of the arginine‐rich domain in Tat‐induced endolysosome dysfunction in human astrocytes. First, we assessed the effect of Tat on endolysosome pH using a ratiometric method; pHLys‐green is pH sensitive, and its fluorescence decreases as pH increases, whereas LysoPrime‐red is not pH sensitive, and its fluorescence does not change as pH increases. Thus, a decreased ratio of pHLys‐green/LysoPrime‐red indicates increased endolysosome pH. Consistent with our previous findings, we demonstrated that Tat (100 nM for 48 h) significantly increased endolysosome pH (endolysosome de‐acidification), as evidenced by a decreased fluorescence ratio of pHLys‐green to LysoPrime‐red (Figure 1G). In contrast, mutant‐Tat failed to induce endolysosome de‐acidification (Figure 1G).

Our previous findings indicate that Tat induces signs of endolysosome membrane leakage (Hui et al. 2012), which could contribute to endolysosome de‐acidification. Thus, we explored the extent to which Tat induces endolysosome membrane leakage, as indicated by the formation of galectin‐3 puncta. Galectin‐3, a cytosolic protein, enters the lumen of endolysosomes and forms puncta when the integrity of the endolysosome membrane is compromised (Aits et al. 2015; Eriksson et al. 2020). Using astrocytes transiently expressing EGFP‐tagged Galectin‐3, we demonstrated that Tat (100 nM for 2 and 24 h), but not mutant Tat, induced the formation of galectin‐3 puncta in human astrocytes (Figure 1H).

Endolysosome damage can lead to augmented release of their luminal contents via exocytosis (Datta et al. 2019; Kim et al. 2021), and in such a process, a variety of factors can be secreted, including cathepsin B (Fan and He 2016; Verderio et al. 2012), ATP (Zhang et al. 2007), exosomes (You et al. 2020), and galectin‐3 (Jia et al. 2020; Popa et al. 2018). Thus, we determined the extent to which Tat induces the secretion of endolysosome luminal contents. We demonstrated that Tat (100 nM for 48 h), but not mutant Tat lacking the arginine‐rich domain, increased the secretion of galectin‐3 (Figure 1I) and cathepsin B (Figure 1J) into the media of human astrocytes. Together, our findings suggest that the arginine‐rich domain is critical for Tat‐induced endolysosome damage.

### 2.2 Tat Interacts With TLR7

The findings above indicate that the arginine‐rich domain appears to be necessary for Tat‐induced endolysosome damage. We postulate that the arginine‐rich domain mediates Tat‐induced endolysosome damage at the site of the endolysosome lumen by interacting with endolysosome‐resident proteins. Thus, we explored the interaction of Tat with endolysosome‐resident proteins, focusing on those proteins that are involved in pathogen sensing in endolysosomes (Miyake et al. 2019).

**FIGURE 2:**
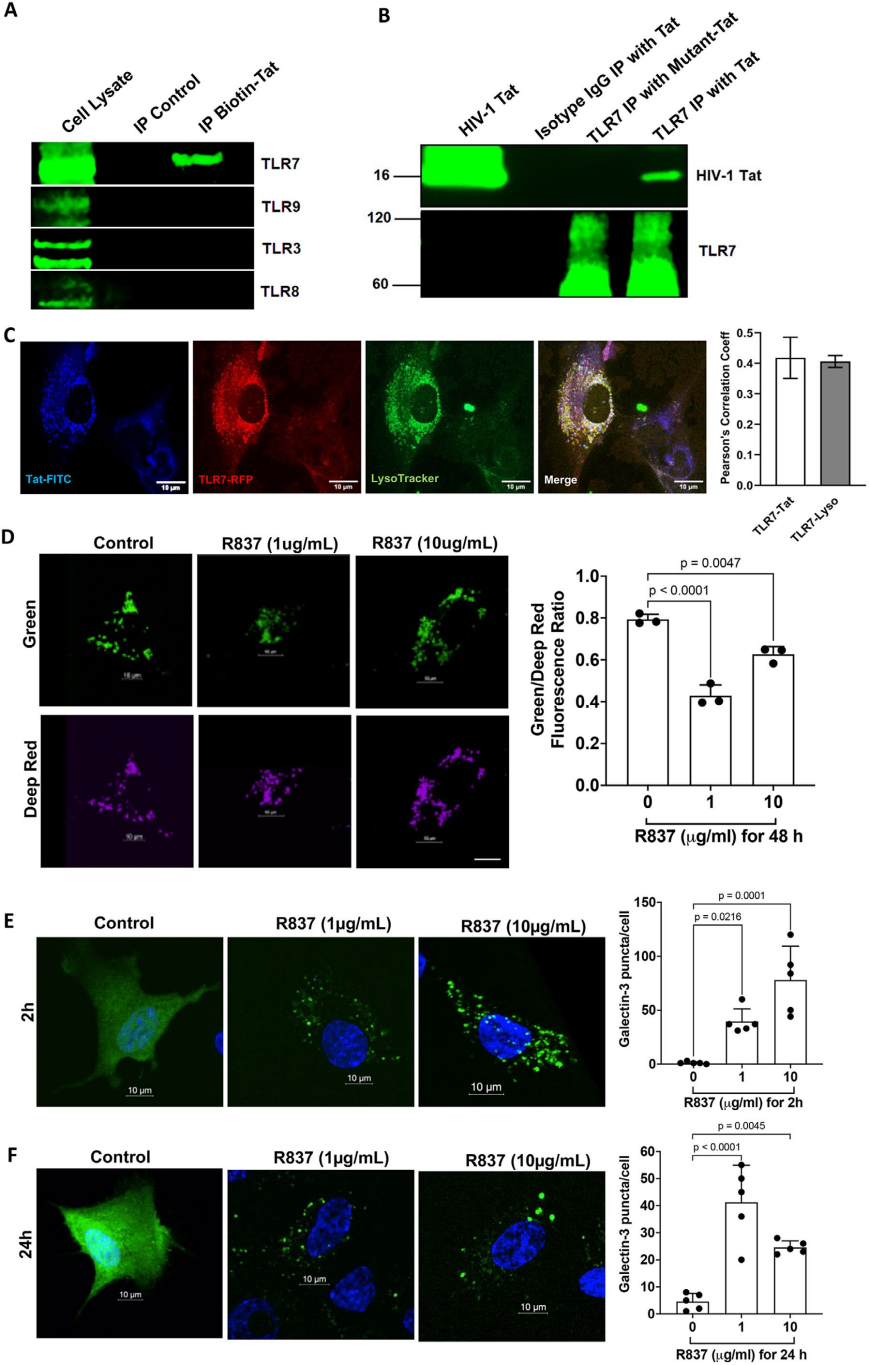
Tat interacts with TLR7, and TLR7 activation induces endolysosome damage. (A) Using biotin‐Tat as a bait, TLR7, but not TLR3, TLR8, or TLR9, was pulled down from U87MG cell lysates. (B) Using a biotinylated anti‐TLR7 antibody as bait to capture TLR7 from U87MG cell lysate and then incubate with Tat or mutant Tat, Tat, but not mutant‐Tat, was detected in the precipitates. A biotinylated isotype IgG was served as a negative control. (C) TLR7‐RFP colocalized with Tat‐FITC (blue) with a Pearson's correlation coefficient of 0.42. TLR7‐RFP colocalized with endolysosomes (Lysotracker, green) with a Pearson's correlation coefficient of 0.4 in human astrocytes (*n* = 3). Scale bar = 10 μm. (D) TLR7 agonist R837 (1–10 μg/mL) induced endolysosome de‐acidification, as indicated by a decreased Green/Deep Red fluorescence ratio, scale bar =10 μm (*n* = *3)*. (E, F) TLR7 agonist R837 (1–10 μg/mL) significantly increased galectin‐3 puncta formation for 2 h (E) and 24 h (F) post‐treatment, scale bar =10 μm (*n* = 5). Statistics: *N* = Independent replicates. One‐way ANOVA followed by Tukey's post hoc test.

As a first step in identifying endolysosome‐resident binding partners of Tat, biotin‐Tat was used as a bait protein to pull down interacting proteins from U87MG cell lysates. The co‐immunocomplexes were separated using SDS‐PAGE and analyzed by immunoblotting with various antibodies targeting endolysosome‐resident TLRs including TLR3, TLR7, TLR8, and TLR9. The immunoblotting results demonstrated that biotin‐Tat pulled down TLR7 (Figure 2A) but not TLR3, TLR8, or TLR9. Next, a biotinylated anti‐TLR7 antibody was used as bait to pull down TLR7 from the U87MG cell lysates; subsequently, the TLR7 co‐immunocomplexes were incubated with either Tat or mutant‐Tat to assess potential interactions between Tat and TLR7. We demonstrated that Tat, but not mutant‐Tat lacking the arginine‐rich domain, interacts with TLR7 (Figure 2B). Thus, the arginine‐rich domain is critical for the interaction between Tat and TLR7.

To further investigate if such an interaction between Tat and TLR7 could happen in endolysosomes of a living cell, we conducted co‐localization studies using live cell imaging. We demonstrated the colocalization of FITC‐Tat with TLR7‐RFP, with a Pearson's correlation coefficient of 0.42, and the colocalization of TLR7 with endolysosomes (Lysotracker), with a Pearson's correlation coefficient of 0.4 (Figure 2C). These results provide strong evidence that Tat interacts with endolysosome‐resident TLR7.

### 2.3 TLR7 Activation Induces Endolysosome Damage in Human Astrocytes

We have shown that Tat induces endolysosome damage in human astrocytes. If Tat induces endolysosome damage via its interaction with TLR7, activation of TLR7 should induce a similar effect. Thus, we investigated the extent to which TLR7 activation affects endolysosome function. First, we examined the impact of the TLR7 agonist R837 on endolysosome pH and endolysosome membrane leakage. We demonstrated that R837 (1–10 μg/mL for 48 h) induced endolysosome de‐acidification in astrocytes, as evidenced by a decreased fluorescence ratio of pHLys‐green to LysoPrime‐red (Figure 2D). Next, we explored the extent to which TLR7 agonist (R837) induces endolysosome membrane leakage as indicated by the formation of galectin 3 puncta. Our results showed that R837 (1–10 μg/mL) significantly increased the formation of galectin‐3 puncta at 2 h and 24 h post‐treatment in human astrocytes (Figure 2E,F).

### 2.4 TLR7 Is Involved in Tat‐Mediated Inflammatory Responses in Human Astrocytes

**FIGURE 3:**
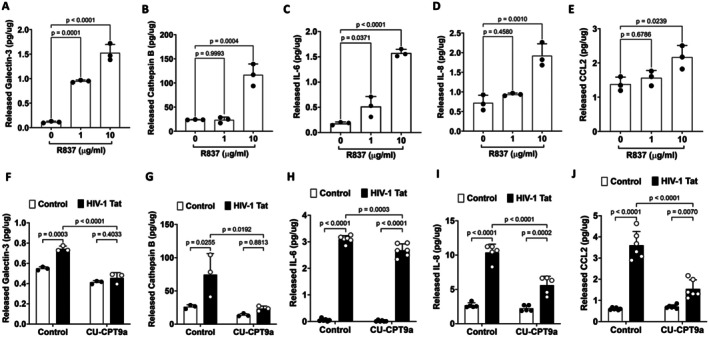
TLR7 is involved in Tat‐induced inflammatory responses in human astrocytes. (A–E) Activation of TLR7 by R837 (0–10 μg/mL for 48 h) significantly increased levels of galectin‐3 (A), cathepsin B (B), IL‐6 (C), IL‐8 (D), and CCL2 (E) in the media of human astrocytes (*n* = 3). (F‐J) Blocking TLR7 with CU‐CPT9a (100 nM for 48 h) alone did not affect the release of inflammatory factors; However, CU‐CPT9a significantly attenuated Tat (100 nM for 48 h)‐induced increased levels of galectin‐3 (F, *n* = *3*), cathepsin B (G, *n* = *3*), IL‐6 (H, *n* = *6*), IL‐8 (I, *n* = *5*), and CCL2 (J, *n* = *6*) in the media of human astrocytes. Statistics: *N* = Independent replicates. One‐way ANOVA with Tukey's post hoc test or two‐way ANOVA with Tukey's post hoc test.

Endolysosome damage can lead to augmented release of their luminal contents via exocytosis, and we have shown that Tat increased the secretion of galectin 3 and cathepsin B, both of which are inflammatory factors that play an important role in neuroinflammatory processes (Arad et al. 2015; Dumic et al. 2006; Mort and Buttle 1997; Nouh et al. 2011; Ramírez et al. 2019; Soares et al. 2021; Srejovic et al. 2020; Tan et al. 2021). Here, we demonstrated that activation of TLR7 with R837, in a concentration‐dependent manner, induces the release of galectin 3 (Figure 3A) and cathepsin B (Figure 3B) in human astrocytes.

It is known that TLR7 activation increases the production of various inflammatory factors including IL‐6 (Vanden Bush and Bishop 2008), IL‐8 (Lu et al. 2023), and CCL2 (Michaelis et al. 2019). If Tat induces the release of inflammatory factors via its interaction with TLR7, activation of TLR7 by its specific agonist should induce a similar inflammatory response. Indeed, we demonstrated that activation of TLR7 with R837, in a concentration‐dependent manner, induces the release of inflammatory factors including IL‐6, IL‐8, and CCL2, in human astrocytes (Figure 3C–E). At the concentrations used, R837 (1–20 μg/mL for 48 h) did not induce cytotoxic effects in human astrocytes as indicated by the released LDH assay (Appendix S1).

Next, we determined the extent to which blocking TLR7 affects the Tat‐induced release of inflammatory mediators. Due to the lack of a specific TRL7 antagonist, we determined the effect of CU‐CPT9a, which exerts an antagonizing effect on TLR7 at higher concentrations (Hu et al. 2018), on Tat‐induced release of inflammatory factors. Using similar concentrations, we have shown that CU‐CPT9a attenuated exogenous Tat‐mediated LTR transactivation (Khan et al. 2022). We demonstrated that CU‐CPT9a co‐treatment (100 nM for 48 h) significantly attenuated Tat‐induced secretion of galectin‐3, cathepsin B, IL‐6, IL‐8, and CCL2 into the media of human astrocytes (Figure 3F–J). At the concentrations used, CU‐CPT9a (0–200 nM for 48 h) did not induce cytotoxic effects in human astrocytes as indicated by the released LDH activity assay (Appendix S1).

**FIGURE 4:**
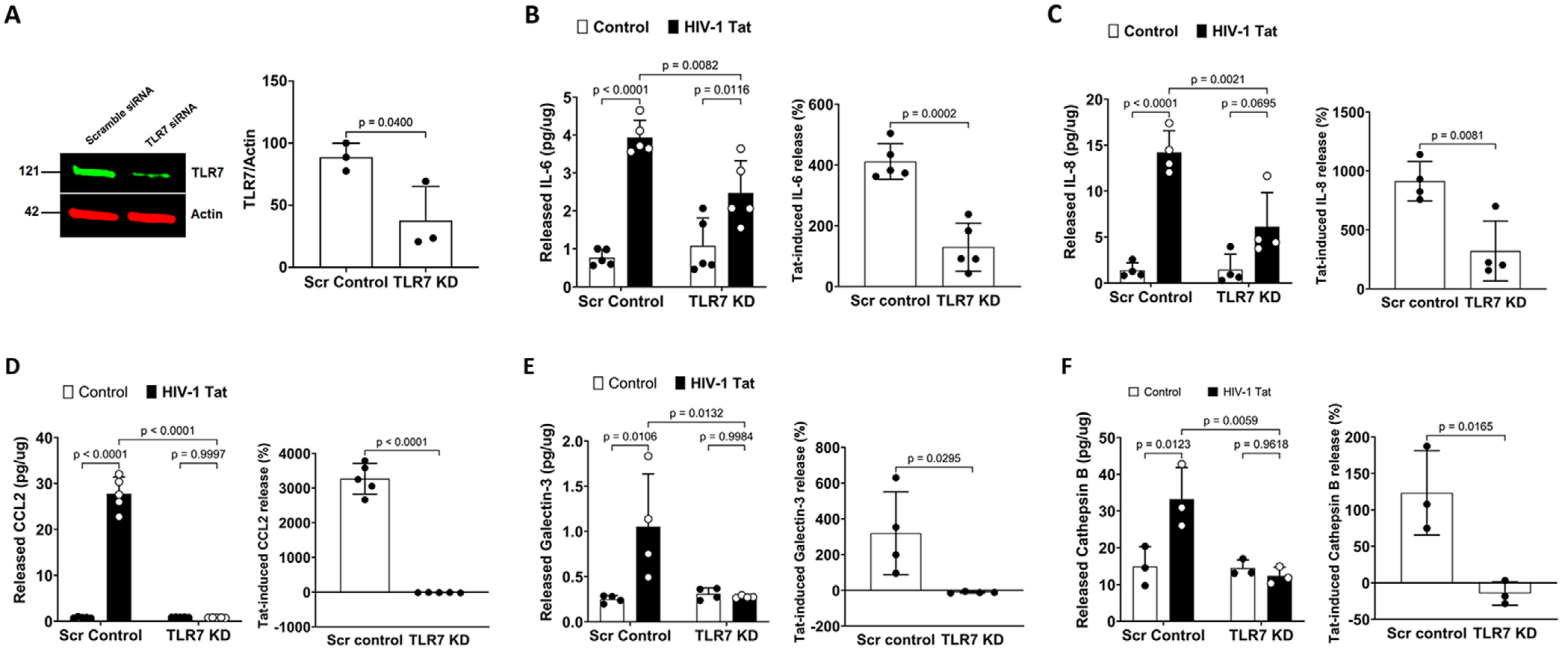
TLR7 knockdown attenuates Tat‐induced inflammatory response in human astrocytes. (A) Quantitative immunoblotting data showed that TLR7 was knocked down with specific siRNAs in human astrocytes (*n* = *3*). (B‐F) TLR7 knockdown alone did not affect the release of these inflammatory factors; However, TLR7 knockdown significantly attenuated Tat‐induced increases in IL‐6 (B, *n* = *5*), IL‐8 (C, *n* = *4*), CCL2 (E, *n* = *5*), galectin‐3 (E, *n* = *4*), and cathepsin B (F, *n* = *3*) in the media of cultured human astrocytes. Statistics: *N* = Independent replicates. Two‐way ANOVA with Tukey's post hoc test and two tailed student's *t*‐test.

To further confirm our pharmacological findings, we determined the extent to which TLR7 knockdown affects Tat‐induced release of inflammatory factors. We demonstrated that TLR7 knockdown (Figure 4A) alone did not affect the release of inflammatory factors; However, TLR7 knockdown significantly attenuated HIV‐1 Tat‐induced release of IL‐6, IL‐8, CCL2, galectin‐3, and cathepsin B into the media of cultured human astrocytes (Figure 4B–F). Together, our findings suggest that Tat induces an inflammatory response via its interaction with endolysosome‐resident TLR7 in human astrocytes.

### 2.5 The Arginine‐Rich Basic Domain Is Critical for Tat‐Induced Cellular Senescence in Human Astrocytes

Cellular senescence is a state of stable cell‐cycle arrest with secretory features in response to various cellular stresses. Endolysosome dysfunction is strongly linked to cellular senescence (Curnock et al. 2023; Gorgoulis et al. 2019; Tan and Finkel 2023), which is associated with dramatic changes in endolysosome structure and function including endolysosome enlargement, endolysosome de‐acidification, endolysosome membrane leakage, and increased endolysosome content, with the SA‐β‐gal being the most widely employed marker of the senescent state. As internalization of Tat represents an early step in exogenous Tat‐induced cellular response, Tat‐induced endolysosome dysfunction could play an upstream role in cellular senescence in astrocytes. In support, we have shown that Tat induces endolysosome damage and increases the release of IL‐6, IL‐8, and CCl2 (Figure 1), all of which are components of SASP (Gonzalez‐Gualda et al. 2021).

**FIGURE 5:**
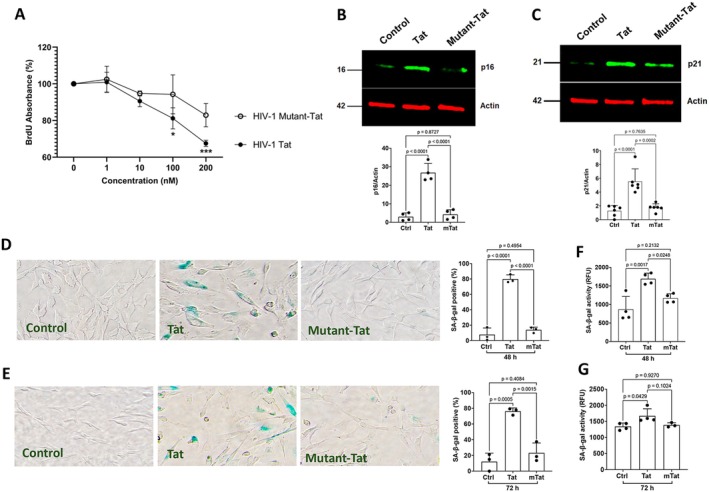
The arginine‐rich domain is critical for Tat‐induced senescence‐like phenotype in human astrocytes. (A) Tat (0–200 nM, 48 h), but not mutant‐Tat lacking the arginine‐rich domain (0–200 nM, 48 h), significantly decreased cell proliferation, as indicated by BrdU incorporation, in human astrocytes (*n* = *3)*. (B, C) Tat (100 nM, 48 h), but not mutant‐Tat, significantly increased protein levels of senescence markers p16^Ink4a^ (B, *n* = *4*) and p21^CIP1^ (C, *n* = *6* Independent replicates) in human astrocytes. (D, E) Tat (100 nM), but not mutant‐Tat (100 nM), significantly increased the percentage of SA‐β‐gal positive cells at 48 h (D) and 72 h (E) post‐treatment (*n* = 3 Independent replicates). (F, G) Tat (100 nM), but not mutant‐Tat (100 nM), significantly increased SA‐β‐gal activity at 48 h (F) and 72 h (G) post‐treatment (*n* = *4*). Statistics: *N* = Independent replicates. One‐way ANOVA with Tukey's post hoc test.

As such, we determined further the extent to which Tat induces a senescence‐like phenotype in astrocytes. Cell cycle arrest is one of the most defining hallmarks of cellular senescence that is characterized by reduced DNA replication and/or cellular proliferation and increased protein markers of cell cycle arrest like p16^Ink4a^ and/or p21^CIP1^. We first assessed the extent to which Tat affects cellular proliferation using a BrdU incorporation assay; BrdU will be incorporated into newly synthesized DNA of actively proliferating cells. We demonstrated that Tat treatment for 48 h at the concentration of 100 nM and above significantly reduced BrdU incorporation (Figure 5A), indicating Tat induces the inhibition of DNA replication and/or cellular proliferation in astrocytes. In contrast, the mutant‐Tat lacking the arginine‐rich domain did not significantly affect BrdU incorporation (Figure 5A). Next, we determined the extent to which Tat affects protein markers of cell cycle arrest. The two major pathways that govern this cell cycle arrest pathway include the p16^Ink4a^/RB and P53/P21^CIP1^ axes. Increased protein levels of p16^Ink4a^ and/or p21^CIP1^ lead to cell cycle arrest. We demonstrated that Tat (100 nM for 48 h), but not mutant‐Tat, significantly increased protein levels of p16^Ink4a^ (Figure 5B) and p21^CIP1^ (Figure 5C).

Another widely used cellular senescence marker is increased senescence‐associated β‐galactosidase (Dimri et al. 1995); increased expression and activity of SA‐β‐gal (a lysosomal hydrolase) indicate increased lysosomal mass. Thus, we examined the extent to which Tat affects the expression and activity of SA‐β‐gal in human astrocytes. Using a colorimetric SA‐β‐gal staining method, we demonstrated that Tat (100 nM), but not mutant‐Tat, significantly increased the percentage of SA‐β‐gal positive cells at 48 h and 72 h post‐treatment (Figure 5D,E). Using a fluorescent‐based SA‐β‐gal activity assay, we also demonstrated that Tat (100 nM), but not mutant‐Tat, significantly enhanced SA‐β‐gal activity at 48 h and 72 h post‐treatment (Figure 5F,G). Thus, the arginine‐rich basic domain is critical for Tat‐induced cellular senescence in human astrocytes.

### 2.6 TLR7 Is Involved in Tat‐Induced Cellular Senescence in Human Astrocytes

**FIGURE 6:**
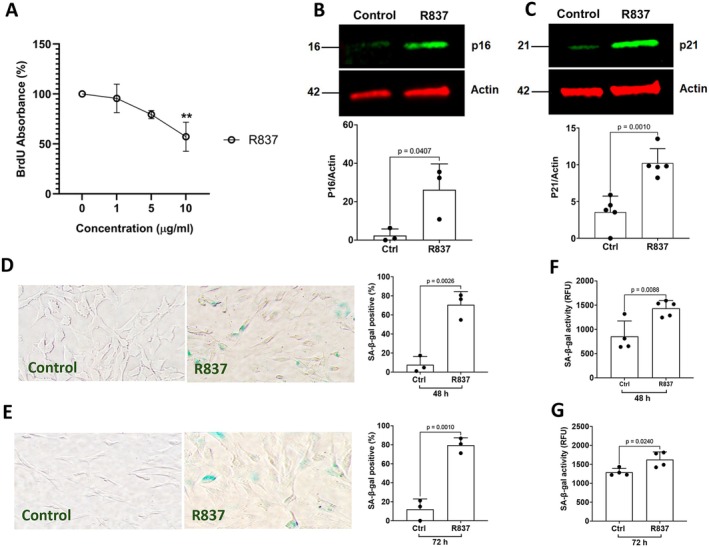
TLR7 activation induces a senescence‐like phenotype in human astrocytes. (A) TLR7 agonist (R837) (10 μg/mL for 48 h) significantly decreased proliferation, as indicated by BrdU incorporation, in human astrocytes *(n = 3)*. (B, C) TLR7 activation with R837 (10 μg/mL for 48 h) significantly increased protein levels of senescence markers p16^Ink4a^ (B, *n* = *3* Independent replicates) and p21^CIP1^ (C, *n* = *5*) in human astrocytes. (D, E) TLR7 agonist R837 (10 μg/mL) significantly increased the percentage of SA‐β‐gal positive cells at 48 h (D) and 72 h (E) post‐treatment (*n* = *3*). (F, G) TLR7 agonist R837 (10 μg/mL), significantly increased SA‐β‐gal activity at 48 h (F) and 72 h (G) post‐treatment (*n* = *4*). Statistics: *N* = Independent replicates. One‐way ANOVA followed by Tukey's post hoc test and two tailed student's *t*‐test.

If Tat induces cellular senescence via its interaction with TLR7, activation of TLR7 by its specific agonist R837 should induce similar effects as that of Tat. We demonstrated that TLR7 agonist R837 significantly reduced BrdU incorporation in a concentration‐dependent manner (Figure 6A), indicating that TLR7 activation inhibits cellular proliferation in human astrocytes. When assessing protein markers of cell cycle arrest, we demonstrated that TLR7 agonist R837 (10 μg/mL) significantly increased protein levels of p16^Ink4a^ (Figure 6B) and p21^CIP1^ (Figure 6C). Furthermore, TLR7 agonist R837 (10 μg/mL) significantly increased the percentage of SA‐β‐gal positive cells at 48 h and 72 h post‐treatment (Figure 6D,E) using a colorimetric SA‐β‐gal staining method. Using a fluorescent‐based SA‐β‐gal activity assay, we also demonstrated that TLR7 agonist R837 (10 μg/mL) significantly enhanced SA‐β‐gal activity at 48 h and 72 h post‐treatment (Figure 6F,G).

**FIGURE 7:**
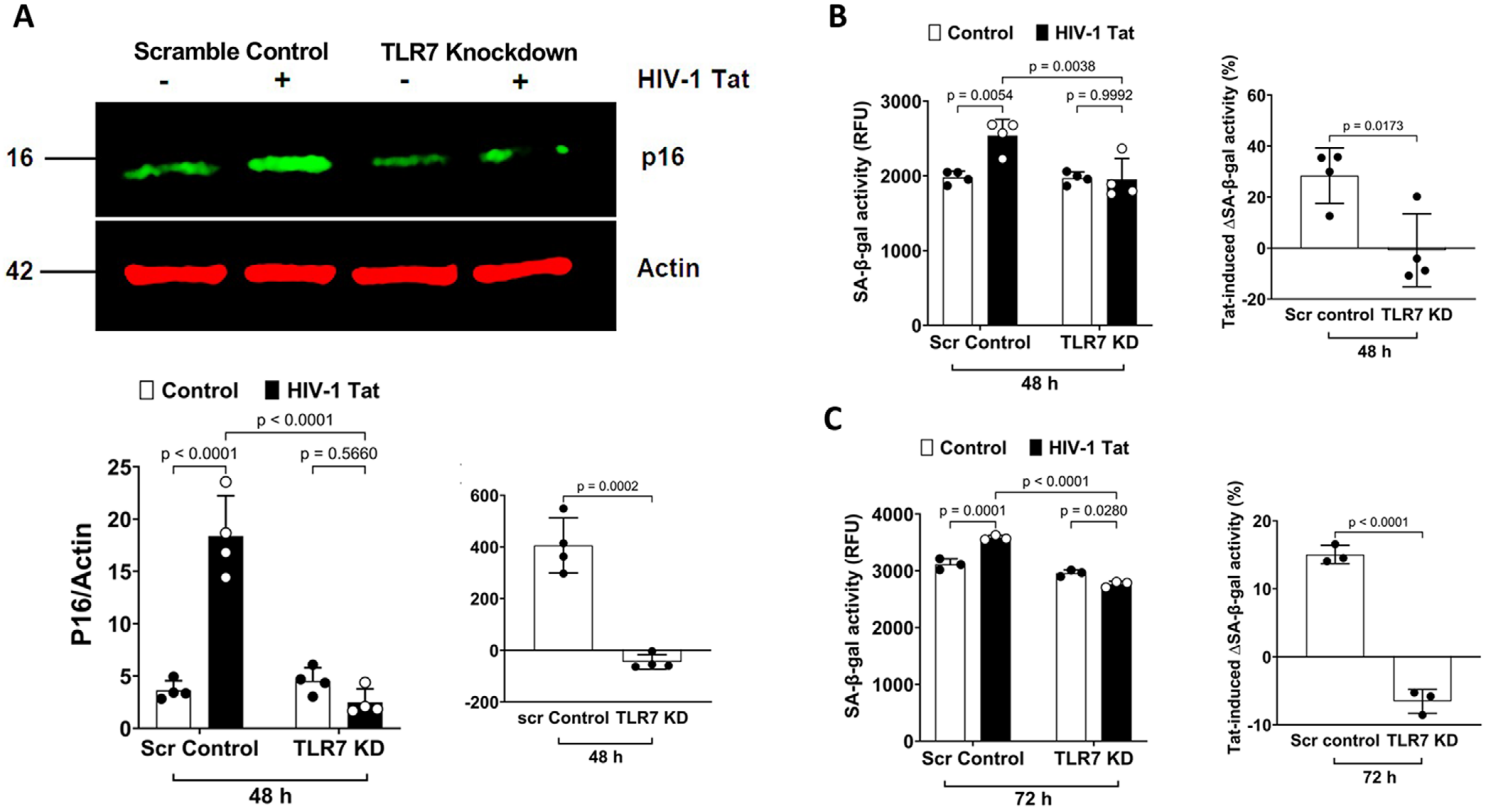
TLR7 knockdown attenuates HIV‐1 Tat‐induced senescence‐like phenotype in human astrocytes. (A) TLR7 knockdown significantly attenuated Tat‐induced increases in p16^Ink4a^ protein levels in human astrocytes (*n* = *4*). (B, C) TLR7 knockdown significantly attenuated Tat (100 nM)‐induced increases in SA‐β‐gal activity at 48 h (B, *n* = *4*) and 72 h (C, *n* = *3*) post‐treatment. Statistics: *N* = Independent replicates. Two‐way ANOVA followed by Tukey's post hoc test and two tailed student's *t*‐test.

Our findings that TLR7 knockdown attenuates Tat‐induced release of SASP (IL‐6, IL‐8, and CCL2) suggest that the interaction between Tat and TLR7 could induce cellular senescence in astrocytes. To confirm such a notion, we determined the extent to which TLR7 knockdown affects Tat‐induced increases in p16 Ink4a protein levels, and we demonstrated that TLR7 knockdown significantly attenuated Tat‐induced increases in p16^Ink4a^ (Figure 7A). We further determined the extent to which TLR7 knockdown affects Tat‐induced increases in SA‐β‐gal activity. TLR7 knockdown alone did not affect the SA‐β‐gal activity; However, TLR7 knockdown significantly attenuated the increase in SA‐β‐gal activity induced by Tat at 48 h (Figure 7B) and 72 h (Figure 7C) post‐treatment. These findings suggest that the interaction between Tat and endolysosome‐resident TLR7 plays a critical role in Tat‐induced cellular senescence in human astrocytes.

## 3 Discussion

Prominent findings of our studies are that the arginine‐rich domain is critical for Tat‐induced endolysosome damage, inflammatory responses, and cellular senescence in human astrocytes. Mechanistically, Tat interacts with endolysosome‐resident TLR7 via the arginine‐rich domain, and such an interaction plays a key role in Tat‐induced endolysosome damage, inflammatory responses, and the senescence‐like phenotype in human astrocytes. Thus, our finding provides mechanistic insights whereby Tat induces endolysosome damage and cellular senescence. We provide compelling evidence that endolysosome damage drives the development of cellular senescence. Our findings also highlight the novel role of TLR7 in the development of cellular senescence.

The development of neuroinflammation in HAND in the ART era is attributed, at least in part, to the persistence of Tat in the brain (Henderson et al. 2019; Johnson et al. 2013; Mediouni et al. 2012), where Tat interacts within neurons, astrocytes, and other CNS cells (Liu et al. 2000). Astrocytes play a critical role in maintaining homeostasis, supporting neuronal function, and modulating immune responses in the CNS (Dong and Benveniste 2001; Giovannoni and Quintana 2020; Haim and Rowitch 2017; Hart and Karimi‐Abdolrezaee 2021; Sidoryk‐Wegrzynowicz et al. 2011; Stobart and Anderson 2013). Astrocytes can be infected by HIV‐1 through cell‐to‐cell contact or endocytosis (Chang et al. 2011; Churchill et al. 2006; Gorry et al. 2003). Due to their abundance and longevity, astrocytes could serve as crucial long‐term reservoirs for HIV‐1 in the brain (McCarthy and Leblond 1988; Sofroniew and Vinters 2010; Vasile et al. 2017). Astrocytes play a significant role in Tat‐induced neuroinflammation in HAND; Tat‐induced astrogliosis and released cytokines and other inflammatory factors (Blanco et al. 2008; Conant et al. 1998; El‐Hage et al. 2005; Henderson et al. 2012; Kutsch et al. 2000; Nath et al. 1999; Priyanka et al. 2020; Tewari et al. 2015; Williams et al. 2009). As such, the focus of the present study is to determine the underlying mechanisms whereby Tat induces astrocyte‐mediated neuroinflammation focusing on the role of endolysosomes. Secreted Tat can enter the endolysosomes of bystander cells via receptor‐mediated endocytosis (Ajasin and Eugenin 2020; Liu et al. 2000; Mann and Frankel 1991; Tyagi et al. 2001; Vendeville et al. 2004), involving the binding of Tat to cell surface receptors such as CD26 (Gutheil et al. 1994; Ohtsuki et al. 2000) and CXC chemokine receptor type 4 (CXCR4) via cysteine‐rich domain (Ghezzi et al. 2000; Secchiero et al. 1999; Xiao et al. 2000), heparan sulfate proteoglycans via basic domain (Argyris et al. 2004; Tyagi et al. 2001; Urbinati et al. 2009), LRP1 via core domain (Cafaro et al. 2024; Chen et al. 2016; Liu et al. 2000), and integrin via the RGD domain (Aurelio Cafaro et al. 2020; Monini et al. 2012; Urbinati et al. 2005a; Urbinati et al. 2005b). Upon entering the endolysosomes, Tat induces endolysosome de‐acidification and dysfunction in neurons and astrocytes (Chen et al. 2013; Hui et al. 2012; Khan et al. 2022).

Endolysosomes, referring to the endosomal‐lysosomal system consisting of endosomes, lysosomes, and autolysosomes, are critical for degrading materials internalized via endocytosis or phagocytosis and intracellular components delivered through autophagy (Bright et al. 2005; Luzio et al. 2007; Mizushima 2007; Mizushima and Komatsu 2011; Otomo et al. 2012), and are essential for metabolism and cellular homeostasis (Ballabio and Bonifacino 2020). Endolysosome dysfunction not only can lead to abnormal accumulation of undegraded materials and subsequent endolysosome enlargement (Datta et al. 2021; Khan et al. 2022), but also can alter endolysosome trafficking (Johnson et al. 2016) and promote the release of their luminal contents into extracellular space via exocytosis (Datta et al. 2019; Kim et al. 2021), which contributes to inflammatory responses (Bordon 2011; Qian et al. 2017; Rawnsley and Diwan 2020; Toyama‐Sorimachi and Kobayashi 2021; Yambire et al. 2019). Significantly, endolysosomes play an important role in the immune responses of astrocytes (Kreher et al. 2021; Li et al. 2008), and endolysosome dysfunction in astrocytes alone leads to neurodegeneration (Di Malta et al. 2012). Furthermore, endolysosome dysfunction is present in post‐mortem brains of HAND patients (Achim et al. 2009; Gelman et al. 2005, 1997; Zhou et al. 2011a). Experimentally, a variety of HIV‐related factors induce endolysosome dysfunction including gp120 (Bae et al. 2014; Datta et al. 2019; Halcrow et al. 2022), Tat (Chen et al. 2013; Fields et al. 2015; Hui et al. 2012), ART drugs (Hui et al. 2021; Tripathi et al. 2019), methamphetamine (Cubells et al. 1994; Talloczy et al. 2008), and morphine (Nash et al. 2019).

Consistent with our previous findings that endolysosome‐resident TLR7 is involved in Tat‐induced HIV‐1 LTR transactivation (Khan et al. 2022), the present studies demonstrate that Tat interacts with TLR7 and induces endolysosome damage via TRL7‐dependent mechanism. Such an observation is consistent with others' findings that activating TLR7 induces endolysosome membrane leakage (Chang et al. 2021). As an endolysosome‐resident pathogen‐associated molecular pattern recognizing protein, TLR7 recognizes single‐stranded RNA (ssRNA) from viruses (Hemmi et al. 2002; Lund et al. 2004). Besides its interaction with ssRNA, TLR7 has been shown to interact with proteins (Tohme et al. 2020). Thus, it is not surprising that we demonstrate the interaction between Tat and TLR7. Furthermore, our findings suggest that such interaction depends on the arginine‐rich basic domain. We have demonstrated that the mutant‐Tat, with the deletion of the arginine‐rich basic domain, enters the endolysosomes of astrocytes but does not induce endolysosome damage. This is likely because the mutant‐Tat, lacking the arginine‐rich basic domain, does not interact with TLR7 to activate TLR7‐dependent pathways.

Activation of TLR7 plays a key role in the innate immune system, triggering the production of pro‐inflammatory cytokines (IL‐6) (Bush and Bishop 2008; Larange et al. 2009; Li et al. 2014; Liu et al. 2013) and chemokines (IL‐8 and CCL2) (Gorden et al. 2005). Given that Tat interacts with TLR7, that activation of TLR7 with R837 increases secretion of IL‐6, IL‐8, and CCL2, and that TLR7 blockage attenuates Tat‐induced secretion of IL‐6, IL‐8, and CCL2, all of which are implicated in the pathogenesis of HAND (AngelaCovino et al. 2016; Yuan et al. 2013), our findings suggest that Tat induces astrocyte‐mediated inflammatory response via its interaction with TLR7. Tat‐induced release of IL‐6 from astrocytes could activate microglia (Gyengesi et al. 2019) and promote microglia pro‐inflammatory functions (Wheeler et al. 2019). Tat‐induced release of IL‐6 and CCL2 could also contribute to the leakage of the BBB (Takeshita et al. 2021; Ye et al. 2024) and the recruitment of perivascular leukocytes into the CNS (Paul et al. 2014; Recasens et al. 2021). Tat‐induced release of IL‐8 and CCL‐2 could lead to collateral damage of oligodendrocytes and oligodendrocyte progenitor cells (Kiray et al. 2016). Furthermore, Tat‐induced release of IL‐6 from astrocytes could induce direct neuronal injury (Sterling et al. 2022) and alter neuronal excitability and synaptic transmission (Zhou et al. 2011b). Besides the release of cytokines and chemokines, the interaction of Tat with TLR7 also induces the release of galectin‐3 and cathepsin B. Extracellular galectin‐3 plays an important role in neuroinflammatory processes (Arad et al. 2015; Dumic et al. 2006; Ramírez et al. 2019; Soares et al. 2021; Srejovic et al. 2020; Tan et al. 2021), and aberrant release of cathepsin B is also implicated in numerous pathological conditions, particularly those involving inflammation and neurodegeneration (Conus and Simon 2010; Man and Kanneganti 2016; Mort and Buttle 1997; Ni et al. 2022; Reiser et al. 2010). Thus, Tat‐induced astrocyte‐mediated inflammatory responses, without compromising cell viability, could sustain a chronic inflammatory state within the CNS and contribute to the chronic neuroinflammation observed in HAND (Conant et al. 1998; Fan and He 2016; Marino et al. 2020; Nookala and Kumar 2014; Zhou and He 2004; Zhou et al. 2004).

Although the underlying mechanisms are not clear, Tat has been shown to induce cellular senescence in various cells (Beaupere et al. 2015; Chen et al. 2017; Hijmans et al. 2018; Thangaraj et al. 2021; Zhan et al. 2016) including astrocytes (Pillai et al. 2023). Cellular senescence is a state of stable cell‐cycle arrest with secretory features in response to various cellular stresses. Recently, endolysosome dysfunction has been strongly linked to cellular senescence (Curnock et al. 2023; Gorgoulis et al. 2019; Tan and Finkel 2023). Profound changes in endolysosome structure and function are found in senescent cells, including endolysosome enlargement, endolysosome de‐acidification, endolysosome membrane leakage, accumulation of lipofuscin, and upregulation of endolysosome enzymes, with the SA‐β‐gal being the most widely employed marker of the senescent state (Kurz et al. 2000; Lee et al. 2006). Thus, the interaction between Tat and TLR7 at the site of the endolysosome not only could result in endolysosome dysfunction but also in the development of cellular senescence. Indeed, we demonstrated that Tat induces a senescence‐like phenotype (cell cycle arrest, increased SA‐β‐gal activity, and enhanced SASP) in human astrocytes. Given that activation of TLR7 with R837 increases the senescence‐like phenotype in astrocytes and that TLR7 knockdown attenuates Tat‐induced senescence‐like phenotype, our findings suggest that Tat induces a senescence‐like phenotype in astrocytes via its interaction with TLR7 at the site of endolysosomes.

The development of cellular senescence in astrocytes not only could result in the loss of their physiological support to neurons, but also could result in the release of SASP that elicits deleterious paracrine‐like effects on neighboring cells such as neurons, contributing to brain aging, neurodegeneration (Holloway et al. 2023; Dos Melo Santos et al. 2024) and cognitive impairment (Csipo et al. 2020; Meldolesi 2023). Thus, our findings that Tat‐induced astrocyte dysfunction and/or senescence‐like phenotype could create a pro‐inflammatory microenvironment that can exacerbate neuronal damage (Cohen et al. 2017; Cohen and Torres 2019; Henderson et al. 2012; Lazic et al. 2022; Lopez‐Teros et al. 2022; Sharma et al. 2011; Tavazzi et al. 2014; Vazquez‐Villasenor et al. 2020), contributing to the development of a chronic inflammatory state (Lazic et al. 2022; Meldolesi 2023), as well as accelerated aging and neurodegeneration in HAND (Cole et al. 2017; Dickens et al. 2017; Mackiewicz et al. 2019; Zhao et al. 2020, 2022).

Together, our findings provided mechanistic insights whereby Tat induces endolysosome damage and cellular senescence. Our findings suggest that endolysosome damage could drive the development of cellular senescence. Our findings underscore the pivotal role of TLR7 in Tat‐induced endolysosome damage, inflammatory responses, and the senescence‐like phenotype in human astrocytes. Such findings provide novel mechanistic insight into the pathogenesis of HAND and highlight TLR7 as a potential therapeutic target for HAND (Dominguez‐Villar et al. 2015; Lederman 2015).

## 4 Limitations

Despite the significant findings of this research, several limitations must be acknowledged. First, we primarily utilized in vitro models of primary human astrocytes to investigate the effects of Tat. While findings from human astrocytes provide valuable insights, they may not fully capture the complex in vivo environment of the human brain. In addition, the interactions between astrocytes and other cell types, such as neurons and microglia, were not investigated. Furthermore, detailed molecular signaling pathways whereby Tat interacts with TLR7 to induce endolysosome dysfunction, inflammation, and senescence were not explored.

## 5 Methods

### 5.1 Cells

Human primary astrocytes (Sciencell, cat#1800) were cultured in *poly*‐*L*‐*lysine coated cell culture plates in* astrocyte medium supplemented with 2% fetal bovine serum (FBS), 1% astrocyte growth supplement (AGS), and 1% penicillin‐streptomycin according to the manufacturer's protocol. Human U87MG cells (ATCC, #HTB‐14) were cultured in Dulbecco's Modified Eagle Medium (DMEM) supplemented with 10% fetal calf serum and 1% penicillin‐streptomycin. Cells were cultured at 37°C in a 5% CO_2_ humidified incubator. Treatment: human astrocytes were treated with various concentrations of recombinant full‐length (1–101) Tat of HIV‐1 (Abcam, cat#ab83353) and/or recombinant mutant‐Tat of HIV‐1 Bal lacking the arginine‐rich domain (ImmunoDX, cat#1062;). Amino acid sequence of Tat from Abcam is MEPVDPNLEP WNHPGSQPKT ACNTCYCKKC SYHCLVCFQT KGLGISYGRK KRRQRRSAPP SSEDHQNPIS KQPLPRTQGD PTGSEESKKK VESKTETDPF D, with a molecular weight of 16kD on PAGE. According to ImmunoDX, the amino acid sequence from 41 to 60 is deleted for recombinant mutant‐Tat, and the amino acid sequence of mutant Tat is MEPVDPKLEP WKHPGSQPKT ACNTCYCKKC CFHCQVCFIT DSQTHQVSLS KQPTSQPAAA PTGPEESKKK VERETETDPV H, with a molecular weight of 14kD on PAGE.

### 5.2 Fluorescent Labeling of Recombinant Mutant‐Tat

The recombinant mutant‐Tat HIV‐1 Bal lacking the arginine‐rich basic domain (ImmunoDX, cat# 1062) was first dialyzed using a Slide‐A‐Lyzer MiNI dialysis device (Thermo Fisher, cat# 69562) with a 7 kDa molecular weight cut‐off membrane to exchange Tris with PBS (pH =7) because the amine‐containing Tris inhibits the labeling. Following dialysis (overnight at 4°C with multiple dialysate exchanges), fluorescent labeling of recombinant mutant‐Tat was conducted using the Alexa Fluor 488 microscale protein labeling kit (Thermo Fisher, cat# A3006) according to the manufacturer's instructions. Briefly, mutant‐Tat (1 mg/mL) and a molar ratio of 2:1 (dye: mutant‐Tat) were used to achieve selective labeling of the amine termini at room temperature for 15 min. Following labeling, conjugate purification was done using the provided spin filter to remove the unconjugated dye.

### 5.3 Live‐Cell Imaging

For assessing internalization of Tat, human astrocytes were incubated with FITC‐labeled HIV‐1 IIIB Tat protein (4 μg/mL, ImmunoDX, cat#1002‐F) or Alexa Fluor 488 labeled HIV‐1 mutant‐Tat (4 μg/mL) and LysoTracker Red DND‐99 (50 nM, Invitrogen cat#L7528) for 1 h at 37°C. For co‐localization of Tat with TLR7, human astrocytes were transfected with a plasmid encoding TLR7‐RFP (Origene, cat#PS100049). 48 h post‐transfection, the cells were incubated with FITC‐labeled HIV‐1 Tat protein (4 μg/mL, ImmunoDX, cat#1002‐F) and LysoTracker Red DND‐99 (50 nM, Invitrogen cat#L7528) for 1 h at 37°C. Following incubation, cells were washed three times with PBS, and fluorescent images were captured using a Zeiss LSM 800 confocal microscope. Images were analyzed with ImageJ software. For Tat internalization, a total of 5 fields under 63X on a Zeiss LSM800 confocal microscope comprising 3–6 cells/field were imaged, and three independent experiments were carried out.

### 5.4 Immunoprecipitation

Pull‐down assay was conducted using EZ‐Link Desthiobiotinylation and Pull‐Down Kit (Thermo Fisher, cat#16138). Briefly, 10 μg of biotinylated Tat HIV‐1 IIIB (ImmunoDX, cat#1002‐B) was added to 50 μL streptavidin agarose resin and incubated for 30 min at room temperature. A nonbiotinylated HIV‐1 Tat served as a negative control. U87MG cells were lysed in NP‐40 lysis buffer (Thermo Scientific, cat#J60766‐AK) with a 1× protease inhibitor cocktail (Thermo Scientific, cat#78441). Following centrifugation at 12,000 g for 20 min at 4°C, the supernatants were collected. After precleaning with streptavidin‐conjugated resins, 100 μg of cell lysates was incubated with resin containing biotinylated bait protein overnight at 4°C. After washing, co‐immunoprecipitants were eluted with a provided elution buffer (Thermo Fisher, cat#16138). Eluted samples were then used for SDS‐PAGE separation and immunoblotting for target proteins including TLR3, TLR7, TLR8, and TLR9, with U87MG cell lysate serving as a positive control.

In a separate immunoprecipitation experiment using a biotinylated protein interaction pull‐down kit (Thermo Fisher cat#21115), 100 μg of cell lysates were first incubated with 30 μg of a biotinylated anti‐TLR7 antibody (Fabgennix, cat#TLR‐701AP) overnight at 4°C with a Biotin‐Rabbit anti‐Mouse IgG secondary antibody (Thermo Fisher, cat#SA5‐10238) as an isotype IgG control. The co‐immunoprecipitation mixture was then incubated with either HIV‐1 Tat or HIV‐1 mutant‐Tat (10 μg protein mixed with 90 μL TBS), overnight at 4°C. After washing, co‐immunoprecipitants were eluted with a provided elution buffer (Thermo Fisher, cat#21115). Eluted samples were then used for SDS‐PAGE gel (4%–12%) separation and immunoblotting for HIV‐1 Tat proteins.

### 5.5 Immunoblotting

Human astrocytes were lysed using 1× RIPA lysis buffer (Thermo Scientific, cat#89900) with a 1× protease inhibitor cocktail (Thermo Scientific, cat#78441). Following centrifugation at 12,000 g for 20 min at 4°C, the supernatants were collected, and protein concentrations were determined using a Bradford protein assay (Bio‐Rad). Proteins (20 μg) were separated by SDS‐PAGE on a 4%–12% gel and subsequently transferred to PVDF membranes using the iBlot 3 dry transfer system (Invitrogen). The membranes were incubated overnight at 4°C with antibodies against target proteins, with actin antibodies used as a control (Abcam, cat#ab179467, dilution 1:3000 and/or Novus, cat#NBP1‐47423, dilution 1:3000). The following antibodies were used: HIV‐1 Tat antibody (ImmunoDX, cat#1302, dilution 1:1000 and/or Santa Cruz, cat#sc‐65,913, dilution 1:250), TLR7 antibody (ABonline, cat#ABIN3021247, dilution 1:250), TLR3 antibody (Thermo Fisher, cat#PA5‐20183, dilution 1:1000), TLR8 antibody (Invitrogen, cat# PA5‐20056, dilution 1:1000), TLR9 antibody (Thermo Fisher, cat# PA5‐20203, dilution 1:1000), p16‐INK4A (Proteintech, cat#10883‐1‐AP, dilution 1:500), and p21^CIP1^ (cell signaling, cat#2947S, dilution 1:500). Following incubation with primary antibodies, blots were developed with corresponding fluorescently conjugated secondary antibodies, including Goat anti‐mouse IgG secondary antibodies (LI‐COR, cat#926‐68070, 926‐32210, dilution 1:5000) and Goat anti‐rabbit IgG secondary antibodies (LI‐COR, cat#926‐32211, 926‐68071, dilution 1:5000). The density of the antibody‐positive protein bands was quantified using a Li‐COR Odyssey Fc Imaging System (LiCor).

### 5.6 Endolysosome pH Measurement

A lysosomal acidic pH detection kit (Dojindo, item code L268‐10) was used to measure endolysosome pH. Human astrocytes were cultured in 35 mm dishes and treated with HIV‐1 Tat (100 nM), HIV‐1 mutant‐Tat (100 nM), or Imiquimod (R837) (InvivoGen, cat#tlrl‐imqs‐1) at 1 μg/mL or 10 μg/mL for 48 h. After treatment, cells were washed twice with serum‐free medium and incubated with LysoPrime Deep Red working solution (1000×) for 30 min at 37°C. Following this, cells were washed again and incubated with pHLys Green working solution (1000×) for an additional 30 min at 37°C. After the final washes, a cell growth medium was added with nuclear stain, and the cells were observed under a confocal microscope. The fluorescence intensities of pHLys Green (excitation at 488 nm, emission at 500–600 nm) and LysoPrime Deep Red (excitation at 633 nm, emission at 640–700 nm) were measured on an Andor DragonFly 200 platform using a cf40 Zyla camera attached to a Leica DMi8 confocal microscope using the Fusion software. Images were exported to tiff format and fluorescence intensity ratios were calculated using the ROI function in ImageJ.

### 5.7 Galectin‐3 Puncta Assay

Human astrocytes were seeded at a density of 15,000 cells per 35 mm dish and transfected the following day with the pEGFP‐hGal3 plasmid (Addgene, cat# 73080), which expresses EGFP‐tagged Galectin‐3. 48 h after transfection, cells were treated with HIV‐1 Tat (100 nM), HIV‐1 mutant‐Tat (100 nM), or Imiquimod (R837) (InvivoGen, cat#tlrl‐imqs‐1) at 1 μg/mL or 10 μg/mL for 2 h and 24 h. Images were acquired on an Andor DragonFly 200 platform using a cf40 Zyla camera attached to a Leica DMi8 confocal microscope using the Fusion software. Images were exported to TIFF format and the puncta counted using Image J. For galectin‐3 puncta assay, a total of 5 fields under 63X on a Zeiss LSM800 confocal microscope comprising 3–6 cells/field were imaged, and five independent experiments were carried out.

### 5.8 LDH Cytotoxicity Assay

An LDH cytotoxicity assay kit (Invitrogen, cat#C20300) was used to assess the cytotoxicity of various reagents on human astrocytes. Briefly, human astrocytes were treated with various concentrations of recombinant full‐length (1–101) Tat of HIV‐1 (Abcam, cat#ab83353), recombinant mutant‐Tat of HIV‐1 Bal lacking the arginine‐rich domain (ImmunoDX, cat#1062), CU‐CPT9a (InvivoGen, cat#inh‐cc9a) or Imiquimod (R837) (InvivoGen, cat#tlrl‐imqs‐1) for 48 h at 37°C, with a 10X lysis buffer as positive control. Following treatment, the cell culture medium was collected, and LDH activity was measured following the provided protocol. The absorbance was measured at 490 nm and 680 nm using a microplate reader (BioTek). Calculated absorbance, by subtracting the 680 nm value from the 490 nm value, was used as relative cytotoxicity.

### 5.9 siRNA Knockdown

For siRNA knockdown of TLR7, target siRNA (50 nM, Dharmacon) and control siRNA (50 nM, Dharmacon) were dissolved in Accell1 transfection media (B‐005000, Dharmacon) and DharmaFECT 1 (T‐2001‐02, Dharmacon) was used as the transfection reagent for human astrocytes. ON‐TARGETplus Human TLR7 siRNA SMARTpool (Dharmacon reagents, horizondiscovery, cat# L‐004714‐00‐0005) and ON‐TARGETplus Non‐targeting Pool (Dharmacon reagents, horizondiscovery, cat# D‐001810‐10‐05) were used as controls. Human astrocytes were transfected for 6 h, followed by treatment with HIV‐1 Tat. Transfected cells were harvested 48 h post‐treatment, and the efficiency of the siRNA‐mediated knockdown was determined with immunoblotting.

### 5.10 Enzyme‐Linked Immunosorbent Assay (ELISA)

The release of inflammatory factors from human astrocytes into the media was quantified using several ELISA kits: Human IL‐6 ELISA kit (Abcam, cat#ab100572), Human IL‐8 ELISA kit (Abcam, cat#ab46032), Proteome profiler human cytokine array kit (R&Dsystem, cat#ARY005B), Human MCP1 ELISA kit (Abcam, cat#ab100586), Human Cathepsin B ELISA kit (Abcam, cat#ab119584), Human Galectin‐3 ELISA kit (Abcam, cat#ab269555), Human IL‐18 ELISA kit (Thermo Fisher, cat#BMS267‐2), Human IFN gamma ELISA kit (Thermo Fisher, cat#KHC4021), Human IL‐1 alpha ELISA kit (Thermo Fisher, cat#BMS243‐2), Human IFN alpha ELISA kit (Thermo Fisher, cat#BMS216), Human IFN beta ELISA kit (R&D system, cat# QK410), Human TNF alpha ELISA kit (Thermo Fisher, cat#BMS223‐4), Human IL‐12 ELISA kit (Abcam, cat#ab46035), Human complement C3 ELISA kit (Abcam, cat#ab108823).

Briefly, following treatment, cell culture supernatants were collected and centrifuged at 1500 g for 2 min to remove cellular debris. Following the manufacturer's protocol, cell culture supernatants in triplicates or standards in duplicates were added to the precoated wells and incubated overnight at 4°C. After washing, biotinylated detection antibodies were added to each well, followed by incubation with HRP‐conjugated streptavidin. TMB substrate was then added, and color development was allowed to proceed for 30 min. The reaction was stopped by the addition of a stop solution, and absorbance was measured at 450 nm using a microplate reader (BioTek). Concentrations of inflammatory factors were determined from standard curves prepared with known concentrations of specific inflammatory factors using a four‐parameter logistic curve fitting in Gen5 software (BioTek Instruments Inc). Concentrations of inflammatory factors were normalized to the total protein content of the cultured cells as determined by the Bradford protein assay.

### 5.11 SA‐β‐Gal Activity Assay

The SA‐β‐gal activity was measured with an SA‐β‐gal activity assay kit (Enzo Life Sciences, cat#ENZ‐KIT129) according to the provided protocol. Briefly, human astrocytes were seeded in 12‐well plates and incubated overnight. Cells were then treated with HIV‐1 Tat (100 nM), HIV‐1 mutant‐Tat (100 nM), and Imiquimod (10 μg/mL) for 48 h and 72 h. Following treatment, cells were lysed with 1× cell lysis buffer and incubated at 4°C for 15 min. The cell lysates were centrifuged at 12,000 g at 4°C for 10 min. The supernatant collected in triplicates was transferred to a 96‐well plate with the addition of 2× assay buffer, and the plate was incubated at 37°C for 3 h in the absence of CO_2_ and protected from light. The reaction was terminated by the addition of a Stop Solution, and fluorescence was measured using a microplate reader (BioTek) at an excitation wavelength of 360 nm and an emission wavelength of 465 nm. The SA‐β‐gal activity was indicated by the relative fluorescent unit (RFU) that was normalized to the total protein content.

### 5.12 SA‐β‐Gal Staining

Staining of β‐Galactosidase was performed using a senescence β‐Galactosidase Staining kit (Cell Signaling, cat# 9860). Briefly, cells were seeded in 12‐well plates and incubated overnight. The next day, cells were treated with HIV‐1 Tat (100 nM), HIV‐1 mutant‐Tat (100 nM), and Imiquimod (10 μg/mL) for 48 h and 72 h. After the treatment period, cells were fixed in 1X fixative solution for 20 min at room temperature. Following PBS washes, the β‐Galactosidase staining solution, adjusted to pH 6.0, was added, and cells were incubated overnight at 37°C in a CO_2_‐free, dry incubator. Cells were then examined under a microscope (Olympus) at 200× magnification for the presence of blue staining, indicating β‐Galactosidase activity. For SA‐β‐gal staining assay, a total of 5 fields under 20× of the Olympus microscope comprising 30–50 cells/field were imaged, and three independent experiments were carried out.

### 5.13 BrdU Cell Proliferation Assay

A BrdU cell proliferation assay kit (MilliporeSigma, cat# QIA58) was used. Human astrocytes were seeded in 24‐well plates and incubated overnight. Cells were then subjected to various treatments including HIV‐1 Tat (0–200 nM), HIV‐1 mutant‐Tat (0–200 nM), Imiquimod (0‐10 μg/mL), and CU‐CPT9a (0–200 nM) for 48 h. During the final 24 h of treatment, a BrdU label (1:2000) was added to the culture media, and cells were incubated for another 24 h. Cells were then fixed, and DNA was denatured using the fixative/denaturing Solution for 30 min at room temperature. After washing, the cells were incubated with an anti‐BrdU antibody for 4 h at room temperature. Following washing, peroxidase goat anti‐mouse IgG HRP conjugate was added and incubated for 45 min. After washing, the chromogenic substrate TMB was added, and the enzymatic conversion of the TMB was visualized calorimetrically. The reaction was stopped, and absorbance was measured at dual wavelengths of 450–540 nm using a microplate reader (BioTek). The absorbance is directly proportional to the amount of BrdU incorporated into the cells, which is an indicator of cell proliferation.

### 5.14 Statistical Analysis

Data were presented as means ± standard deviations. For comparisons between two groups, statistical significance was determined using two‐tailed Student's *t*‐test. Comparisons among multiple groups with one factor were conducted using one‐way ANOVA followed by Tukey's post hoc test to adjust for multiple comparisons. Comparisons among multiple groups with two factors were conducted using two‐way ANOVA followed by Tukey's post hoc test to adjust for multiple comparisons. A *p*‐value of less than 0.05 was considered statistically significant.

## Author Contributions

N.R. and X.C. designed the research. N.R., G.D., W.A.H., E.C.N., and E.V.S. performed experiments and analyzed data. N.R. drafted the manuscript, and X.C. revised the manuscript.

## Consent

This article does not contain any studies with human participants performed by any of the authors.

## Conflicts of Interest

The authors declare no conflicts of interest.

## Supporting information


Appendix S1.

## Data Availability

The data that support the findings of this study are available on request from the corresponding author. The data are not publicly available due to privacy or ethical restrictions.
