# Supplementary material for: TLR7 Mediates HIV‐1 Tat‐Induced Cellular Senescence in Human Astrocytes
Source: Aging Cell. 2025 Apr 30;24(7):e70086. doi: 10.1111/acel.70086 (PMC12266786; doi:10.1111/acel.70086)
Supplement: Supplementary file 1 — Appendix S1. [file ACEL-24-e70086-s001.docx]

Supplementary Data


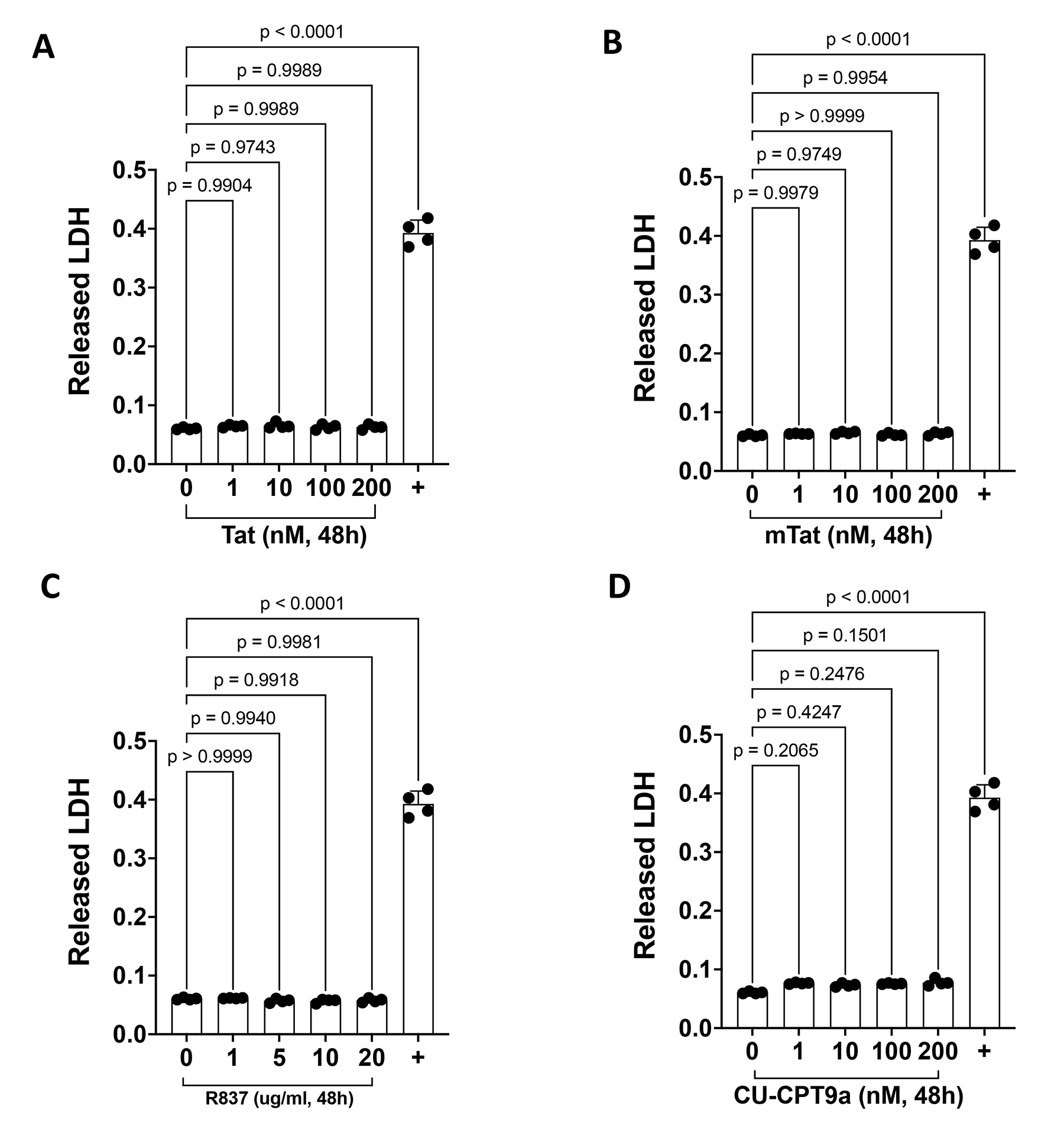


**Supplementary Figure. Cytotoxicity as indicated by released LDH activity.**

(A) Tat (0-200 nM for 48 h) did not induce cytotoxic effects in human astrocytes as indicated by released LDH activity *(n=4)*. Released LDH activity by cell Lysis solution was used as a positive control (+). (B) mutant Tat (mTat, 0-200 nM for 48 h) did not induce cytotoxic effects in human astrocytes as indicated by released LDH activity *(n=4)*. Released LDH activity by cell Lysis solution was used as a positive control (+). (C) TLR7 agonist R837 (0-20 μg/ml for 48 h) did not induce cytotoxic effects in human astrocytes as indicated by released LDH activity *(n=4)*. Released LDH activity by cell Lysis solution was used as a positive control (+). (D) CU-CPT9a (0-200 nM for 48 h) did not induce cytotoxic effects in human astrocytes as indicated by released LDH activity *(n=4)*. Released LDH activity by cell Lysis solution was used as a positive control (+). Statistics: One-way ANOVA followed by Tukey's post hoc test.
